# Chronic Idiopathic Neutrophilia in Two Twins

**DOI:** 10.1155/2014/785454

**Published:** 2014-11-06

**Authors:** Roberto Miniero, Giuseppe Antonio Mazza, Federica Altomare, Carla Fusaro

**Affiliations:** ^1^Pediatric Department, “Magna Graecia” University of Catanzaro, Località Germaneto, Viale Europa, 88100 Catanzaro, Italy; ^2^Primary Pediatric Care, ASP Cosenza, Italy

## Abstract

Neutrophilia in adults refers to an alteration in the total number of blood neutrophils that is in excess of about 7500 cells/*μ*L. This definition is restrictive in childhood as neutrophil count is age-dependent. Chronic Idiopathic Neutrophilia (CIN) refers to a condition that persists for many years in individuals who appear otherwise healthy. CIN is rarely mentioned in scientific literature and in academic books of hematology; only few words are dedicated to this topic. We report a case study of two twins with CIN followed from the first year of life to 24 years of age. To the best of our knowledge this is the first case report of two twins with CIN followed through a long period of time. We believe that our observation may contribute to better understand and characterize this hematologic abnormality.

## 1. Introduction

Neutrophilia in adults refers to an alteration in the total number of blood neutrophils that is in excess of about 7500 cells/*μ*L. This definition is restrictive in childhood as neutrophil count is age-dependent: leukocyte neutrophils are high at birth and during the first few days of life, and then a decrease to adult levels occurs within the first few weeks and thereafter is maintained during the childhood. An increase in circulating neutrophils is the result of a disturbance of the normal steady-state involving neutrophil bone marrow production, movement in and out of the marrow compartments into the circulation, and neutrophil destruction. In particular three mechanisms, either alone or in combination, largely account for neutrophilia. First, there may be expansion of the circulating neutrophil pool as a result of increased progenitor cell proliferation and terminal differentiation through the neutrophilic series.

Second, increased numbers of neutrophils may be mobilized from either the bone marrow storage compartment or the peripheral marginating pools into the circulating pool. Finally, there may be increased blood neutrophil survival owing to impaired neutrophil egress into tissue [1].

The clinical significance of neutrophilia is uncertain. When approaching a patient with neutrophilia we must consider many factors as the degree of neutrophil count, additional hematologic abnormalities, presence and duration of clinical symptoms, and comorbid conditions. The hematologic and nonhematologic diseases and conditions associated with neutrophilia are listed below [2].

*Classification of Neutrophilia*

*Increased Production*

 Chronic infection
 Chronic inflammation
   Ulcerative colitis
   Rheumatoid arthritis
 Tumors (perhaps with necrosis)
 Postneutropenia rebound
 Myeloproliferative disease
 Drugs (lithium, occasionally ranitidine)
 Familial cold urticarial
 Leukemoid reactions
 Chronic Idiopathic Neutrophilia

*Enhanced Release from Marrow Storage Pool*

 Corticosteroids
 Stress
 Hypoxia and smoke
 Acute infection
 Endotoxin

*Decreased Egress from Circulation*

 Corticosteroids
 Splenectomy and asplenia
 Leukocyte adhesion deficiency

*Reduced Margination*

 Stress
 Infections
 Exercise
 Epinephrine

Chronic neutrophilia may follow the prolonged administration of glucocorticoids, persistent inflammatory reactions, infections, chronic blood loss, or chronic anxiety. Most reactions of this type last for days or weeks, but some may persist for many months. Pyogenic microorganisms, leptospiral infection, and certain viruses (including herpes simplex, varicella, rabies, and poliomyelitis) may produce neutrophilia. Significant neutrophilic leukocytosis has also been reported with both Kawasaky disease and infectious mononucleosis. Occasionally, extreme neutrophilia has been observed in tuberculosis, usually in seriously ill patients with widespread necrotizing inflammatory disease. Chronic inflammation is often responsible for persistent neutrophilia, especially in patients with juvenile rheumatoid arthritis. Sustained moderate neutrophilia invariably follows either surgical or functional asplenia; it probably arises because of a failure to remove circulating neutrophils (a normal function of the spleen) rather than because of an increase in granulopoiesis. Neutrophilia was also reported in functional disorders of neutrophils associated with impaired adhesion or motility, such as that found in patients with leukocyte adhesion deficiency or actin dysfunction.

Instead Chronic Idiopathic Neutrophilia (CIN) refers to a condition that persists for many years in individuals who appear otherwise healthy without any underlying diseases or conditions listed above and it has been reported, until now, almost exclusively in adult age. CIN is rarely mentioned in scientific literature and in academic books of hematology; only few words are dedicated to this topic. The most extensive contribution to the definition of CIN is a recent study by Weir and colleagues who reported a cohort of 134 adult patients with leukocyte count between 11000 and 40000/mm^3^ without other clinical or hematologic comorbidities, except for the fact that many of them were smokers [3].

## 2. Case Presentation

We report a case study of two twins with CIN followed from the first year of life to 24 years of age. Written informed consent was obtained from the patients to present this case report.

**Figure 1:**
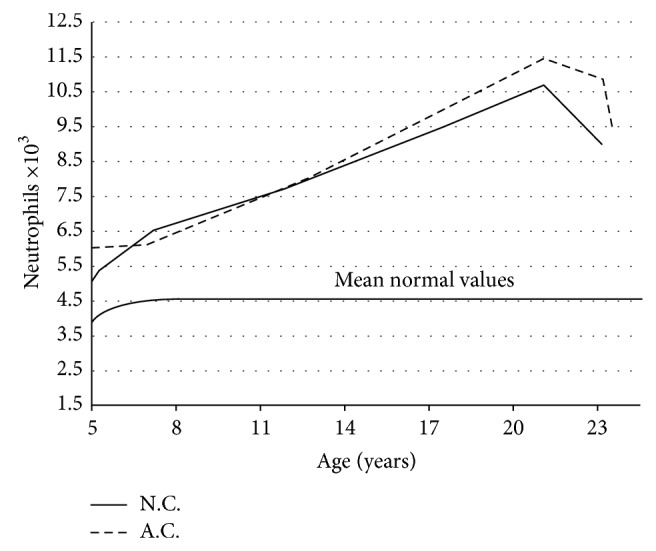
Neutrophil levels of the two patients during the follow-up.

Two monochorionic female twins of Caucasian origin presented for the first time at the age of 24 years for a persistent neutrophilia without clinical symptoms. Since the early years of life till the age of 9 neutrophil leukocytes at the complete blood count, performed every year, have been higher compared to the average reference values for age; thereafter they have further increased to a true neutrophilia (more than 2SD from mean values for age) (Figure 1).

Granulocyte morphology was normal without immature forms in the peripheral smear. The other leucocyte subpopulations, red blood cells, and platelets parameters have remained normal throughout the period of observation. Hematologic parameters were normal in parents.

All causes of neutrophilia reported in the list mentioned in Section 1 were ruled out. The patients have had an overall good health during their life; in particular they have never had persistent inflammations or infections; they disclaimed prolonged administration of glucocorticoids or lithium; spleen size has always been normal for age; finally they had no other risk factors for neutrophilia such as obesity and/or smoking. Then, owing to these clinical and laboratory conditions, diagnosis of CIN has been formulated at the time of our observation.

## 3. Discussion

Chronic neutrophilia is a rare condition in pediatrics. When approaching a patient with such condition it is very important to focus the attention on his history and physical examination, to perform a complete blood count, a chemistry profile including liver function tests, a chest X-ray, other laboratory and imaging assays based on identified abnormalities, and a blood smear that has to be reviewed by a hematologist or pathologist. Finally focused history and physical examination, complete blood count, and chemistry profile must be repeated at 2, 6, and 12 months and annually thereafter. Additional testing, including PCR or FISH techniques for bcr-abl, JAK-2 mutational testing, erythropoietin levels, and bone marrow examination with cytogenetics, must be warranted for patients suspected of having a myeloproliferative neoplasm [3].

To the best of our knowledge this is the first case report of two twins with CIN followed through a long period of time. The diagnosis has been performed according to the criteria suggested by the literature: persistent isolated increase of neutrophil leucocytes in subjects otherwise healthy without any underlying diseases and conditions listed in previous list mentioned in Section 1. For these reasons we decided not to perform other additional hematologic tests in order to minimize the burden of diagnostic insights on young patients who were otherwise healthy.

The underlying mechanism leading to CIN remains still unknown. In 2009 Plo and colleagues identified the first case of a germ line activating mutation in the CSF3R gene in a family with CIN associated with splenomegaly, based on the autosomal-dominant pattern of inheritance. This mutation energetically favours dimerization of the colony-stimulating factor (G-CSF) receptor transmembrane domain and thus strongly promotes constitutive activation of the receptor and hypersensitivity to G-CSF for proliferation and differentiation, which ultimately leads to chronic neutrophilia. The survey of 12 affected individuals during three generations indicates that only one patient had a myelodysplastic syndrome. The authors suggested that mutations in the CSF3R gene might be responsible for hereditary neutrophilia mimicking a myeloproliferative disorder. Of note all patients had splenomegaly and this does not agree with the definition of CIN according to which patients might be asymptomatic at all. Authors also investigated for this mutation other 40 patients with sporadic unexplained neutrophilia but they did not find other positive cases, suggesting that CSF3R mutation is very rare [4]. Evans and colleagues, investigating the genetic and environmental determinants of variation in blood cell number in 392 pairs of 12-year-old twins, demonstrated that the basal levels of white blood cells are genetically regulated with enormous interindividual variation [5]. In our two twins the trends of neutrophilia have been stackable during the entire period of observation. This agrees with the hypothesis that in these cases CIN might be related to a germinal genetic mutation that modifies the normal pattern of neutrophil kinetics and confirms the genetic influence for total neutrophil count during development.

With the exception of the family reported by Plo, where a mutation of a single gene produced a condition of CIN transmitted by an autosomal-dominant character, no other cases of genetic forms have been described until now. In our case parents were normal so we might suggest a form of CIN with an autosomal recessive transmission or a* de novo mutation*. We believe that our observation may contribute to better understand and characterize this hematologic abnormality.
